# The Activin A-Peroxisome Proliferator-Activated Receptor Gamma Axis Contributes to the Transcriptome of GM-CSF-Conditioned Human Macrophages

**DOI:** 10.3389/fimmu.2018.00031

**Published:** 2018-01-29

**Authors:** Concha Nieto, Rafael Bragado, Cristina Municio, Elena Sierra-Filardi, Bárbara Alonso, María M. Escribese, Jorge Domínguez-Andrés, Carlos Ardavín, Antonio Castrillo, Miguel A. Vega, Amaya Puig-Kröger, Angel L. Corbí

**Affiliations:** ^1^Centro de Investigaciones Biológicas, Consejo Superior de Investigaciones Científicas (CSIC), Madrid, Spain; ^2^Instituto de Investigación Sanitaria, Fundación Jiménez Díaz, Madrid, Spain; ^3^Instituto de Investigación Sanitaria Gregorio Marañón, Hospital General Universitario Gregorio Marañón, Madrid, Spain; ^4^Centro Nacional de Biotecnología, Consejo Superior de Investigaciones Científicas (CSIC), Madrid, Spain; ^5^Instituto Investigaciones Biomédicas “Alberto Sols” (IIBM), and Centro Mixto Consejo Superior de Investigaciones Científicas y Universidad Autónoma de Madrid (ICSIC-UAM), Madrid, Spain; ^6^Unidad de Biomedicina (Unidad Asociada al CSIC), IIBM-Universidad Las Palmas de Gran Canaria (ULPGC), and Instituto Universitario de Investigaciones Biomédicas y Sanitarias (IUIBS), Universidad Las Palmas de Gran Canaria (ULPGC), Las Palmas de Gran Canaria, Spain

**Keywords:** transcription factor, macrophage, peroxisome proliferator-activated receptor, inflammation, innate immunity

## Abstract

GM-CSF promotes the functional maturation of lung alveolar macrophages (A-MØ), whose differentiation is dependent on the peroxisome proliferator-activated receptor gamma (PPARγ) transcription factor. In fact, blockade of GM-CSF-initiated signaling or deletion of the PPARγ-encoding gene *PPARG* leads to functionally defective A-MØ and the onset of pulmonary alveolar proteinosis. *In vitro*, macrophages generated in the presence of GM-CSF display potent proinflammatory, immunogenic and tumor growth-limiting activities. Since GM-CSF upregulates PPARγ expression, we hypothesized that PPARγ might contribute to the gene signature and functional profile of human GM-CSF-conditioned macrophages. To verify this hypothesis, PPARγ expression and activity was assessed in human monocyte-derived macrophages generated in the presence of GM-CSF [proinflammatory GM-CSF-conditioned human monocyte-derived macrophages (GM-MØ)] or M-CSF (anti-inflammatory M-MØ), as well as in *ex vivo* isolated human A-MØ. GM-MØ showed higher PPARγ expression than M-MØ, and the expression of PPARγ in GM-MØ was found to largely depend on activin A. Ligand-induced activation of PPARγ also resulted in distinct transcriptional and functional outcomes in GM-MØ and M-MØ. Moreover, and in the absence of exogenous activating ligands, PPARγ knockdown significantly altered the GM-MØ transcriptome, causing a global upregulation of proinflammatory genes and significantly modulating the expression of genes involved in cell proliferation and migration. Similar effects were observed in *ex vivo* isolated human A-MØ, where PPARγ silencing led to enhanced expression of genes coding for growth factors and chemokines and downregulation of cell surface pathogen receptors. Therefore, PPARγ shapes the transcriptome of GM-CSF-dependent human macrophages (*in vitro* derived GM-MØ and *ex vivo* isolated A-MØ) in the absence of exogenous activating ligands, and its expression is primarily regulated by activin A. These results suggest that activin A, through enhancement of PPARγ expression, help macrophages to switch from a proinflammatory to an anti-inflammatory polarization state, thus contributing to limit tissue damage and restore homeostasis.

## Introduction

Tissue-resident macrophages in homeostasis, as well as monocyte-derived macrophages within inflamed tissues, exhibit a huge functional diversity which derives from their exquisite sensitivity to extracellular cues (1, 2). GM-CSF and M-CSF drive macrophage differentiation and survival (3). However, M-CSF is required for the generation of most tissue macrophages (4, 5) while GM-CSF is needed for development and maintenance of pulmonary alveolar macrophages (A-MØ) (6). Besides its role in myeloid cell differentiation, GM-CSF is a central mediator of tissue inflammation (7) and its neutralization has been proposed as a therapeutic strategy for inflammatory disorders (8). As a consequence, both colony-stimulating factors promote the generation of functionally distinct macrophages (9): GM-CSF-conditioned human monocyte-derived macrophages (GM-MØ) produce large amounts of proinflammatory cytokines in response to stimulation, whereas M-CSF-dependent monocyte-derived macrophages (M-MØ) primarily produce anti-inflammatory factors upon activation (9–11). At the transcriptional level, while GM-MØ are characterized by the expression of a “*Proinflammatory gene set*” (11–13) also detected in macrophages under inflammatory conditions *in vivo*, M-MØ specifically express an “*Anti-inflammatory gene set*” and resemble macrophages from homeostatic/anti-inflammatory settings (14, 15). Interestingly, the GM-MØ-specific gene signature is critically determined by activin A both *in vivo* (15) and *in vitro* (11). In this regard, we have previously demonstrated that GM-MØ produce large amounts of activin A, a member of the TGFβ family (16, 17) that regulates inflammatory responses (18), modulates cytokine release (19, 20) and myeloid cell differentiation (21), and whose functional blockade in GM-MØ skews cells toward the acquisition of an anti-inflammatory signature (11).

The contribution of GM-CSF to differentiation of lung macrophages relies on the GM-CSF-dependent expression of peroxisome proliferator-activated receptor gamma (PPARγ) (22–24), a nuclear receptor that regulates gene transcription through ligand binding (25–28), antagonism of other transcription factors (e.g., NFκB, AP-1) (29, 30) and recruitment of repressor complexes in the absence of ligands (31). As a critical regulator of inflammatory processes (32–34), PPARγ inhibits human and murine macrophage responses to proinflammatory stimuli (35, 36), contributes to IL-4-driven polarization of human and murine macrophages (37, 38) and determines the acquisition of the metabolic disease-specific phenotype of human macrophages (39). In human cells, the ubiquitously expressed PPARγ1 derives from the *PPARG1* and *PPARG3* mRNA splicing isoforms, while the *PPARG2* mRNA isoform codes for PPARγ2, whose expression is restricted to adipocytes (40). Mouse A-MØ exhibit much higher expression of PPARγ than other macrophages in the steady-state (34), and its GM-CSF-dependent expression is essential for their differentiation and maturation from fetal monocytes (24). In fact, PPARγ expression in A-MØ is lost in GM-CSF-deficient mice and in patients with pulmonary alveolar proteinosis (PAP), a pathology derived from a defective expression or activity of GM-CSF (23, 41) and associated with suppressed activin A expression (42). However, it is currently unknown whether PPARγ is required for maintenance of A-MØ throughout adult life (43).

Upon tissue injury, monocyte-derived macrophages modulate inflammation and also promote tissue repair. In the specific case of lung inflammation, monocyte-derived mouse A-MØ are the major drivers of fibrosis and become similar to tissue-resident A-MØ over time (44). Since GM-CSF-conditioned monocyte-derived human macrophages exhibit potent proinflammatory functions upon stimulation (9, 11), and in spite of the intrinsic anti-inflammatory functions of PPARγ, we hypothesized that PPARγ might contribute to the gene signature and functional profile of human GM-CSF-conditioned macrophages. To address this hypothesis, we evaluated the extent of the PPARγ contribution to the gene signature and functional profile of human GM-CSF-dependent macrophages. We now report the activin A-dependent expression and activity of PPARγ in GM-CSF-conditioned human macrophages, and demonstrate that PPARγ displays polarization-dependent activities and significantly shapes the gene signature of proinflammatory monocyte-derived GM-MØ and human A-MØ in the absence of exogenous ligands. The activin A-dependent expression of PPARγ in GM-MØ and in A-MØ also suggests a role for activin A in promoting inflammation resolution.

## Experimental Procedures

### Generation of Human Monocyte-Derived Macrophages *In Vitro* and *Ex Vivo* Isolation of A-MØ

Buffy coats were obtained from healthy blood donors, as anonymously provided by the Comunidad de Madrid blood Bank. Ethical approvals for all blood sources and processes used in this study were approved by the Centro de Investigaciones Biológicas Ethics Committee. All experiments were carried out in accordance with the approved guidelines and regulations. Human PBMCs were isolated from buffy coats over a Lymphoprep™ gradient (#1114545, Axis-Shield PoC AS) according to standard procedures. Monocytes were purified from PBMCs by magnetic cell sorting using human CD14 microbeads (#130-050-201, Miltenyi Biotech). Monocytes (95% CD14^+^ cells) were cultured at 0.5 × 10^6^ cells/ml for 7 days in RPMI 1640 (#21875-034, Gibco) supplemented with 10% inactivated fetal calf serum (FCS) (#S1810-500, Biowest) (complete medium), at 37°C in a humidified atmosphere with 5% CO_2_, and containing 1,000 U/ml human GM-CSF (#11343125, Immunotools GmbH) or 10 ng/ml human M-CSF (#11343115, Immunotools GmbH), to generate GM-MØ or M-MØ, respectively. Cytokines were added every 2 days. Blocking anti-activin A Ab (100 ng/ml) (#MAB3381, clone 69403, R&D Systems) or the inhibitors of ALK4, ALK5, and ALK7, SB431542 (10 µM) (#S4317, Sigma-Aldrich) or A-83 (1 µM) (#2039, Tocris) were added every 24 h. Finally, polarized macrophages were treated with ultrapure *Escherichia coli* 0111:B4 strain LPS (10 ng/ml) (#tlrl-3pelps, Invivogen) for 14–16 h. Exposure to recombinant human activin A (25 ng/ml) (#120-14P, Preprotech) was done for 24 h (monocytes and THP-1 cells) or 48 h (M-MØ). The acute monocytic leukemia cell line THP-1, obtained from ATCC^®^ (#TIB-202™), was cultured in complete medium at 37°C in a humidified atmosphere with 5% CO_2_. A-MØ were obtained from patients undergoing bronchoalveolar lavage (BAL) following the Fundación Jiménez Díaz Medical Ethics committee procedures and after written informed consent from all subjects, in accordance with the Declaration of Helsinki. BAL procedure was performed with a flexible bronchoscope with a total volume of 200 ml of sterile isotonic saline solution at 37°C. BAL fluid fractions were maintained at 4°C and cellular debris removed using a 40 µm cell strainer (45). BAL cells were washed with PBS, centrifuged and resuspended in complete medium containing 100 U/mL penicillin and 100 µg/mL streptomycin (#15140-122, Gibco), 50 µg/ml gentamicin (#G1397, Sigma-Aldrich), and 2.5 µg/ml amphotericin B (#A2942, Sigma-Aldrich). The cells were seeded at 6–8 × 10^5^ cells per well in 12-well plates for 1 h and washed extensively to remove non-adherent cells. Finally, 2 ml of complete medium with antibiotics was added to each well and the adherent cells incubated for 16–18 h before transfection. More than 95% of adherent BAL cells were identified as macrophages according to morphology and phenotypic analysis.

### Generation of Murine Bone Marrow-Derived Macrophages *In Vitro*

All experiments on mice were conducted according to the Spanish and European regulations on care and protection of laboratory animals and were approved by the Centro de Investigaciones Biológicas animal facility and the Consejo Superior de Investigaciones Científicas Ethics Committee. Bone marrow-derived GM-MØ or M-MØ were obtained by flushing the femurs of 6–10-week-old C57BL/6 mice (provided by the Animal facility at the Centro de Investigaciones Biológicas), and culturing cells during 7 days in DMEM (#41966-029, Gibco) supplemented with 10% FCS and 50 mM 2-ME, containing either murine GM-CSF (1,000 U/ml) (#315-03, PreProtech) or human M-CSF (25 ng/ml) (#11343115, Immunotools GmbH), respectively (46, 47). Cytokines were added every 2 days.

### Flow Cytometry

Mouse monoclonal antibodies specific for human CD14 (Alexa Fluor-647-labeled antihuman CD14, #301818, clone M5E2, Biolegend) and human CD163 (PerCP-labeled antihuman CD163, #333625, clone GHI/61, Biolegend) were used. Isotype-matched PerCP-labeled Mouse IgG1 (κ Isotype Ctrl Antibody, #400147, clone MOPC-21, Biolegend) and Alexa Fluor-647 Mouse IgG2a (κ Isotype Ctrl Antibody, #400234, clone MOPC-173, Biolegend) were included as negative controls.

### Quantitative Real Time RT-PCR

Total RNA was extracted using the total RNA and protein isolation kit (Macherey-Nagel). RNA samples were retrotranscribed with the High-Capacity cDNA Reverse Transcription kit (AB), and individually amplified cDNA was quantified using the Universal Human Probe Roche library (Roche Diagnostics). Oligonucleotides for selected genes were designed according to the Roche software for quantitative real-time PCR (qRT-PCR), and their sequence is indicated in Table S1 in Supplementary Material. qRT-PCR was performed on a LightCycler^®^ 480 (Roche Diagnostics). Assays were made in triplicates, and results were normalized according to the expression levels of *TBP* mRNA or/and *GAPDH* mRNA (for qRT-PCR) or to the mean of the expression level of endogenous reference genes *HPRT1, TBP* and *RPLP0* (for microfluidic gene cards). Results were expressed using the ΔΔCT (cycle threshold) method for quantification.

### ELISA

Macrophage supernatants were tested for the presence of cytokines using commercially available ELISA sets for human TNFα (BD OptEIA Human TNF ELISA set, #555212, BD Biosciences), CCL2 (BD OptEIA Human MCP-1 ELISA set, #555179, BD Biosciences), IL-10 (ELISA MAX Standard set, #430601, BioLegend), IL-6 (ELISA MAX Standard set, #430501, BioLegend), and activin A (DuoSet, #DY338, R&D Systems), following the protocols supplied by the manufacturers.

### Cell Transfection and Reporter Gene Assays

HEK293-T cells, provided by the Cell culture facility at the Centro de Investigaciones Biológicas, were transfected with an expression vector for PPARγ2 (pBABE-PPARγ2, Addgene) or an empty vector using Superfect transfection reagent (#301305, Qiagen). Human GM-MØ or M-MØ (1 × 10^6^ cells) were transfected using the Human Macrophage Nucleofector^®^ Kit (#VPA-1008, Lonza) with 1 µg of PPAR reporter DNA mixture (#CCS-3026L, Cignal PPAR Reporter assay kit, Qiagen). This mixture contains a PPAR-responsive firefly luciferase construct and a constitutively expressing *Renilla* luciferase (40:1) The PPAR-dependent construct encodes the firefly luciferase gene under the control of a minimal CMV promoter and tandem repeats of the PPAR responsive element (PPRE). Firefly and *Renilla* luciferase activities were determined by using the Dual-Luciferase^®^ Reporter Assay System (#E1910, Promega).

### Western Blot Assay

Cell lysates (40 µg) and nuclear extracts (30 µg) were subjected to SDS-PAGE and transferred onto an Immobilon polyvinylidene difluoride membrane (Millipore, Bedford, MA, USA). After blocking the unoccupied sites with 5% nonfat dry milk, protein detection was carried out with a goat polyclonal against PPARγ2 (G-18, #sc-22020, Santa Cruz Biotechnology), a goat affinity purified polyclonal antibody against Sp1 (PEP2, #sc-59-G, Santa Cruz Biotechnology), or a monoclonal antibody against GAPDH (6C5, #sc-32233, Santa Cruz Biotechnology), and using the SuperSignal West Pico Chemiluminescent system (#34081, Thermo Fisher Scientific).

### Small Interfering Ribonucleic Acid (siRNA) Transfection

To silence *PPARG* gene expression, human GM-MØ, M-MØ (1 × 10^6^ cells) or A-MØ (6–8 × 10^5^ cells) were transfected with a *PPARG*-specific siRNA (siPPARG) (50 nM) (#s10888, Thermo Fisher Scientific), using HiPerFect transfection reagent (#301705, Qiagen). A negative control siRNA from the same company was used as a transfection control (siControl) (#4390843, Thermo Fisher Scientific). After 6 h of transfection, cells were allowed to recover from transfection in RPMI 1640 medium with 10% FCS and the cells were treated with GW7845 (1 µM) (kindly provided by Jon Collins, Glaxo SmithKline, USA) or DMSO for 18–24 h before assessing for PPARγ markers.

### Microarray Analysis

Global gene expression analysis was performed on RNA obtained from three independent samples of GM-MØ that had been transfected with siPPARG or siControl for 48 h, and using a whole human genome microarray from Agilent Technologies (Palo Alto, CA, USA). Only probes with signal values >60% quantile in at least one condition were considered for the differential expression and statistical analysis. Statistical analysis for differential gene expression was carried out using empirical Bayes moderated t test implemented in the limma package^1^ and using paired *t*-test. All the above procedures were coded in R.^2^ Microarray data were deposited in the Gene Expression Omnibus^3^ under accession no. GSE88768. The differentially expressed genes were analyzed for annotated gene sets enrichment using the online tool ENRICHR^4^ (48, 49). Enrichment terms were considered significant when they had a Benjamini-Hochberg-adjusted *p* value < 0.05. For gene set enrichment analysis (GSEA) (50), the previously defined “Proinflammatory gene set” and “Anti-inflammatory gene set” (12), which contain the top and bottom 150 probes from the GM-MØ versus M-MØ limma analysis of the microarray data in GSE68061 (ranked on the basis of the value of the *t* statistic), were used.

### Statistical Analysis

Statistical analysis was performed using paired Student’s *t*-test, and *p* < 0.05 was considered significant (*p < 0.05, **p < 0.01, and ***p < 0.001).

## Results

### PPARγ Activation Has Different Transcriptional and Functional Outcomes in Human GM-MØ and M-MØ

**Figure 1:**
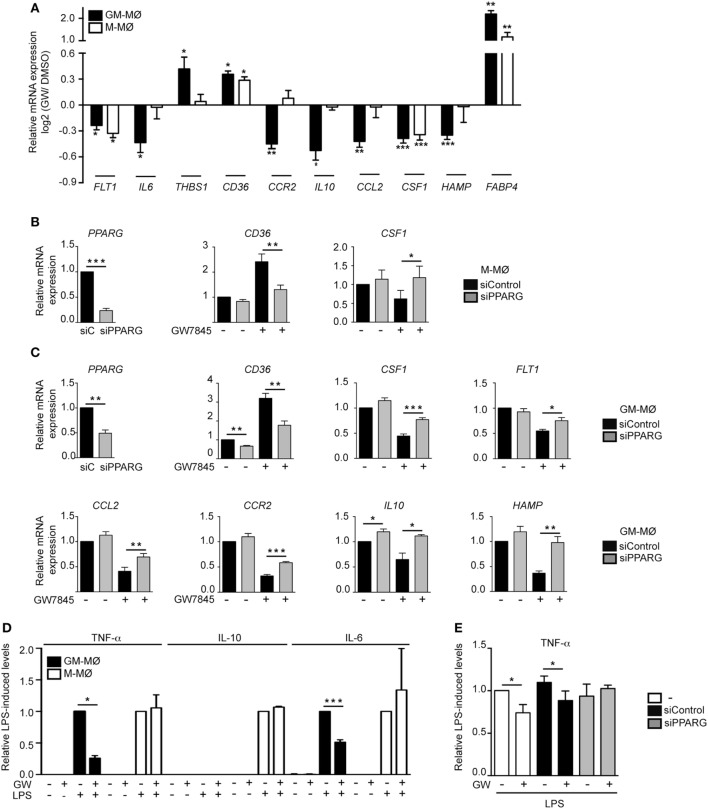
Peroxisome proliferator-activated receptor gamma (PPARγ) mediates the differential effect of GW7845 on the gene and cytokine profile of human GM-CSF-conditioned monocyte-derived macrophages (GM-MØ) and M-CSF-dependent monocyte-derived macrophages (M-MØ). **(A)** Expression of the indicated genes in GM-MØ and M-MØ exposed for 24 h to either GW7845 (GW, 1 µM) or vehicle (DMSO), as determined by quantitative real-time PCR assay using microfluidic gene cards. Results are indicated as the expression of each gene after GW7845 treatment relative to its expression in the presence of DMSO. Each experiment was performed in triplicate, and mean and SEM of three independent experiments is shown (*p < 0.05; **p < 0.01; ***p < 0.001). **(B,C)** Expression of the indicated genes in M-MØ **(B)** or GM-MØ **(C)** transfected with either siPPARG or siControl (*siC*), and treated with GW7845 (1 µM) or DMSO for 24 h. Relative mRNA expression indicates the expression of each gene in the different conditions and relative to its expression in DMSO-treated *siC*-transfected cells (arbitrarily set to 1). (*Left panels*) *PPARG* mRNA expression in siPPARG-transfected cells relative to the *PPARG* mRNA level in *siC*-transfected cells (arbitrarily set to 1). Mean and SEM of four independent experiments are shown (*p < 0.05; **p < 0.01; ***p < 0.001). **(D)** TNFα, IL-10, and IL-6 production in LPS-treated (24 h) GM-MØ and M-MØ that had been preexposed (4 h) to DMSO or GW7845 (GW, 1 µM). Results indicate the concentration of each cytokine for each condition relative to the cytokine levels detected in cells treated with DMSO and LPS (arbitrarily set to 1). Mean and SEM of three independent experiments are shown (*p < 0.05; ***p < 0.001). **(E)** TNFα production in LPS-treated (24 h) untransfected (−), siControl-transfected or siPPARG-transfected GM-MØ that had been preexposed (4 h) to DMSO or GW7845 (GW, 1 µM). Results indicate the concentration of TNFα for each condition relative to the cytokine levels detected in untransfected cells treated with DMSO and LPS (arbitrarily set to 1). Mean and SEM of four independent experiments are shown (*p < 0.05).

**Figure 2:**
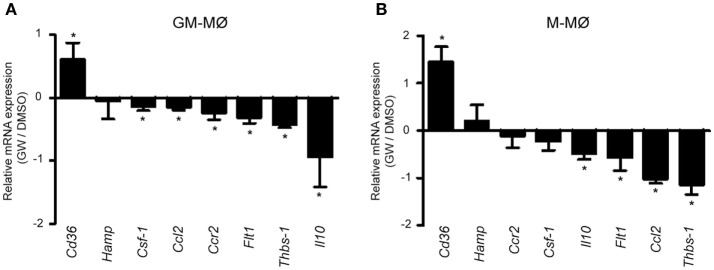
Differential effect of peroxisome proliferator-activated receptor gamma activation on mouse GM-CSF-conditioned bone marrow-derived macrophages (GM-MØ) and M-CSF-dependent bone marrow-derived macrophages (M-MØ). Relative expression of the indicated genes in murine bone marrow-derived GM-MØ (**A**) and M-MØ (**B**) exposed to either DMSO or GW7845 (1 µM) for 24 h, as determined by quantitative real-time PCR. Results are expressed as the expression of each gene in the presence of GW7845 relative to its expression in the presence of vehicle (DMSO). Mean and SD of three independent experiments is shown (*p < 0.05).

To initially assess the PPARγ activation-dependent transcriptional profile of GM-MØ and M-MØ, both human macrophage subtypes were exposed for 24 h to the PPARγ agonist GW7845 and the expression of the GM-MØ-specific “*Proinflammatory gene set*” and M-MØ-specific “*Anti-inflammatory gene set*” (derived from the data contained in the Gene Expression Omnibus GSE68061) (11, 12) was determined. PPARγ activation upregulated the paradigmatic PPARγ target genes *CD36* and *FABP4*, and downregulated *FLT1* and *CSF1* expression, in both macrophage subtypes (Figure 1A). However, GW7845 downregulated *IL*6, *IL10, CCL2, HAMP*, and *CCR2* and enhanced *THBS1*, exclusively in GM-MØ (Figure 1A). These GW7845-triggered gene expression changes were dependent on PPARγ activation as they were significantly impaired upon siRNA-mediated knockdown of *PPARG* mRNA (Figures 1B,C). Specifically, *PPARG* mRNA knockdown inhibited the GW7845-mediated modulation of *CD36* and *CSF1* expression in M-MØ (Figure 1B) and significantly impaired the GW7845-mediated modulation of *CD36, CSF1, FLT1, CCL2, CCR2, IL10*, and *HAMP* in GM-MØ (Figure 1C). Analogous findings were observed in murine bone marrow-derived macrophages, where Pparγ activation modified the expression of a common set of genes in both macrophage subtypes but significantly diminished the expression of *Csf1* and *Ccr2* only in GM-MØ (Figure 2). Therefore, although PPARγ activation alters the expression of known PPARγ targets in both GM-MØ and M-MØ, it also promotes human macrophage subtype-dependent transcriptional changes because the expression of *CCR2, IL10, CCL2*, and *HAMP* is downregulated by GW7845 only in proinflammatory GM-MØ.

To determine whether the distinct transcriptional effects of PPARγ activation in GM-MØ and M-MØ had a functional correlate, the LPS-induced cytokine-producing ability of both macrophage subtypes was evaluated in the presence of GW7845. As expected, LPS stimulation of GM-MØ caused the preferential production of the proinflammatory cytokines TNFα and IL-6, whereas LPS-stimulated M-MØ primarily released IL-10 (9–11) (Figure 1D). In line with the transcriptional results, GW7845 significantly reduced the LPS-induced production of TNFα and IL6 from GM-MØ, but had no effect on the LPS-induced cytokine release from M-MØ (Figure 1D). Importantly, the inhibitory effect of GW7845 on the LPS-induced TNFα production of GM-MØ was PPARγ-dependent, as it was reduced upon PPARγ knockdown (Figure 1E). Therefore, agonist-mediated activation of PPARγ exclusively modulates the LPS-induced cytokine production from proinflammatory human monocyte-derived GM-MØ, further arguing for a polarization-dependent effect of PPARγ in human macrophages.

### PPARγ Is Preferentially Expressed by Proinflammatory GM-CSF-Dependent Human Macrophages

**Figure 3:**
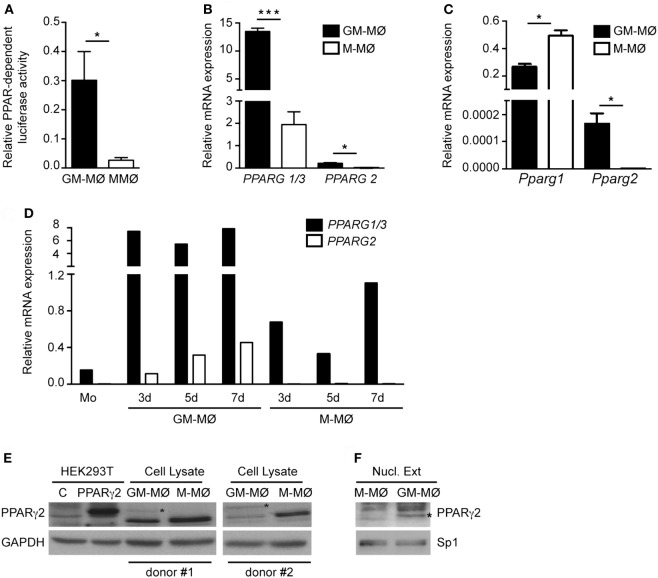
Expression of peroxisome proliferator-activated receptor gamma (PPARγ) isoforms in human and mouse GM-CSF-conditioned macrophages (GM-MØ) and M-CSF-dependent macrophages (M-MØ). **(A)** Basal PPAR-dependent transcriptional activity in GM-MØ and M-MØ. Mean and SEM of the relative PPAR-dependent luciferase activity (compared to *Renilla* luciferase activity) of seven independent experiments is shown (*p < 0.05). **(B)** *PPARG1/3* and *PPARG2* mRNA expression levels in GM-MØ and M-MØ, as determined by quantitative real-time PCR (qRT-PCR) and relative to *TBP* mRNA levels. Mean and SEM of three independent experiments is shown (*p < 0.05; ***p < 0.001). **(C)** *Pparg1* and *Pparg2* mRNA expression in bone marrow-derived murine GM-MØ and M-MØ, as determined by qRT-PCR and relative to *Tbp* mRNA levels. Mean and SD of three independent samples is shown (*p < 0.05). **(D)** *PPARG1/3* and *PPARG2* mRNA expression levels along GM-MØ and M-MØ differentiation, as determined by qRT-PCR and relative to *TBP* mRNA levels. A representative experiment is shown. **(E,F)** PPARγ2 protein levels in whole cell **(E)** or nuclear lysates (Nucl. Ext.) **(F)** from mock-transfected (C) or human PPARγ2-transfected HEK293T cells **(E)** and two independent samples of GM-MØ and M-MØ (donor #1 and donor #2). GAPDH **(E)** and Sp1 **(F)** protein levels were detected in parallel as protein loading controls. The band corresponding to PPARγ2 protein is indicated by an asterisk.

Given the different effect of PPARγ on GM-MØ and M-MØ, we next determined PPARγ expression and function in both human macrophage subtypes. Transfection of a PPRE reporter construct in both macrophage subtypes revealed that global PPAR-dependent transcriptional activity is higher in GM-MØ than in M-MØ (Figure 3A), thus suggesting that GM-MØ are endowed with a stronger PPARγ-dependent transcriptional activity. Regarding expression, GM-MØ contained higher levels of *PPARG1/3* (encoding the ubiquitous PPARγ1 isoform) and *PPARG2* (coding for the PPARγ2 isoform) mRNAs than M-MØ (Figure 3B). In fact, the adipocyte-restricted *PPARG2* mRNA (40) was barely detectable in M-MØ (Figure 3B). The preferential expression of the PPARγ2-encoding mRNA was also observed in murine bone marrow-derived GM-MØ, whereas mouse M-MØ exhibited significantly higher *Pparg1* expression than mouse GM-MØ (Figure 3C), in agreement with a previous report (51) and in line with the distinct gene profiles of monocyte-derived human M-MØ and bone marrow-derived mouse M-MØ (13, 52). Kinetic analysis revealed that *PPARG2* mRNA is upregulated in human monocytes exposed to GM-CSF for 3, 5, and 7 days (Figure 3D). Although *PPARG1* is expressed at higher levels than *PPARG2* mRNA (25-fold approx.), PPARγ2 protein could be detected in whole cell and nuclear extracts from GM-MØ (Figures 3E,F). Therefore, GM-CSF-conditioned proinflammatory human macrophages exhibit a higher expression of PPARγ (PPARγ1 and PPARγ2) than M-CSF-conditioned anti-inflammatory human macrophages.

### Activin A Controls PPARγ Expression in GM-CSF-Dependent Macrophages

**Figure 4:**
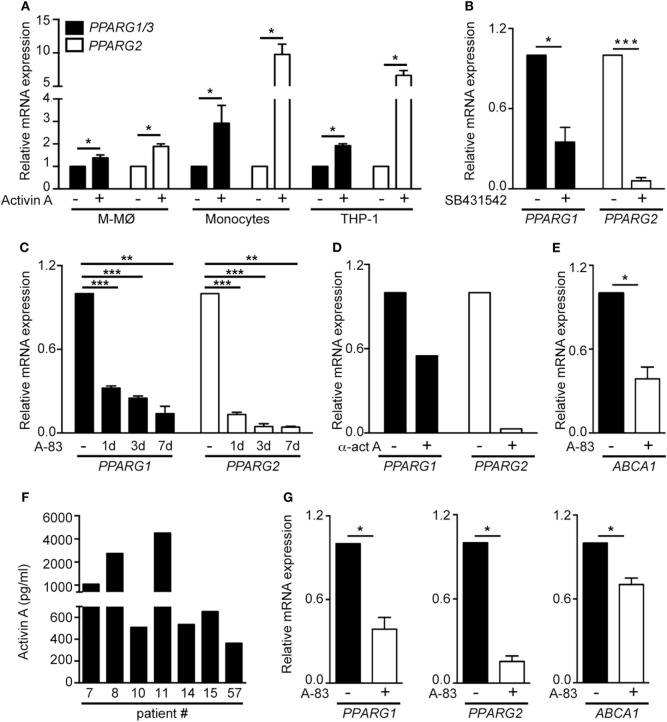
The activin A/Smad signaling pathway determines the differential expression of *PPARG1* and *PPARG2* in human GM-CSF-conditioned monocyte-derived macrophages (GM-MØ) and M-CSF-dependent monocyte-derived macrophages (M-MØ). **(A)** *PPARG1* and *PPARG2* expression in untreated or activin A-treated M-MØ, monocytes and THP-1 cells. Cells were treated with 25 ng/ml recombinant human activin A for 24 h (monocytes and THP-1 cells) or 48 h (M-MØ). Mean and SEM of three independent experiments is shown (*p < 0.05). **(B)** *PPARG1* and *PPARG2* gene expression in GM-MØ generated in the presence of either DMSO or the Smad signaling inhibitor SB431542 (10 µM). Mean and SEM of three independent experiments is shown (*p < 0.05; ***p < 0.001). **(C)** *PPARG1* and *PPARG2* gene expression in GM-MØ generated in the presence of either DMSO or the Smad signaling inhibitor A-83 (1 µM) for 1, 3, or 7 days. Mean and SEM of three independents experiments is shown (*p < 0.05; **p < 0.01; ***p < 0.001). **(D)** *PPARG1* and *PPARG2* gene expression in GM-MØ generated in the presence of a neutralizing antiactivin A antibody (α-ActA) or an isotype-matched antibody (−). One representative experiment is shown. In **(A–D)**, results are referred to the *PPARG1* or *PPARG2* mRNA levels detected in untreated cells (arbitrarily set to 1). **(E)** *ABCA1* gene expression in GM-MØ generated in the presence of either DMSO or the Smad signaling inhibitor A-83 (1 µM) for seven days. Results are referred to the *ABCA1* mRNA level in DMSO-treated cells. Mean and SEM of three independents experiments is shown (*p < 0.05). **(F)** Activin A expression levels in seven independent samples of human alveolar macrophages kept in culture for 24 h after isolation. **(G)** *PPARG1, PPARG2*, and *ABCA1* mRNA levels in human alveolar macrophages cultured for 24 h after isolation in the presence of either DMSO or the Smad signaling inhibitor A-83 (1 µM). Results are referred to the mRNA levels of each gene in DMSO-treated cells (arbitrarily set to 1). Means and SEM of three independents samples is shown (*p < 0.05).

The GM-CSF-dependent expression of PPARγ is essential for the differentiation of A-MØ (24). Since proinflammatory human GM-MØ polarization is dependent on the autocrine/paracrine action of activin A (11), we next questioned whether activin A contributes to the preferential expression of *PPARG* mRNAs in GM-MØ. Activin A significantly elevated *PPARG1/3* and *PPARG2* mRNA levels in M-MØ, monocytes and THP-1 myeloid cells (Figure 4A). Moreover, inhibition of activin A-initiated Smad signaling by either SB431542 (Figure 4B) or A-83 (Figure 4C), or blockade of activin A with an anti-activin neutralizing antibody (Figure 4D), significantly reduced *PPARG1/3* and *PPARG2* mRNA levels in GM-CSF-dependent proinflammatory GM-MØ. In line with these results, generation of GM-MØ in the presence of A-83 resulted in significantly reduced expression of the PPARγ target gene *ABCA1*, a gene whose expression is responsive to PPARγ-LXR activation in human macrophages (Figure 4E). Further, analysis of *ex vivo* isolated human A-MØ revealed the constitutive expression of activin A (Figure 4F), and that the expression of *PPARG1/3* and *PPARG2* mRNA, as well as the expression of the PPARγ target *ABCA1* mRNA, were significantly reduced in the presence of the A-83 Smad signaling inhibitor (Figure 4G). Altogether, these results indicate that activin A is a positive regulator of PPARγ expression and activity in GM-CSF-conditioned macrophages both *in vitro* and *in vivo*.

### Identification of the PPARγ-Dependent Gene Profile in GM-CSF-Conditioned Proinflammatory Human Macrophages

**Figure 5:**
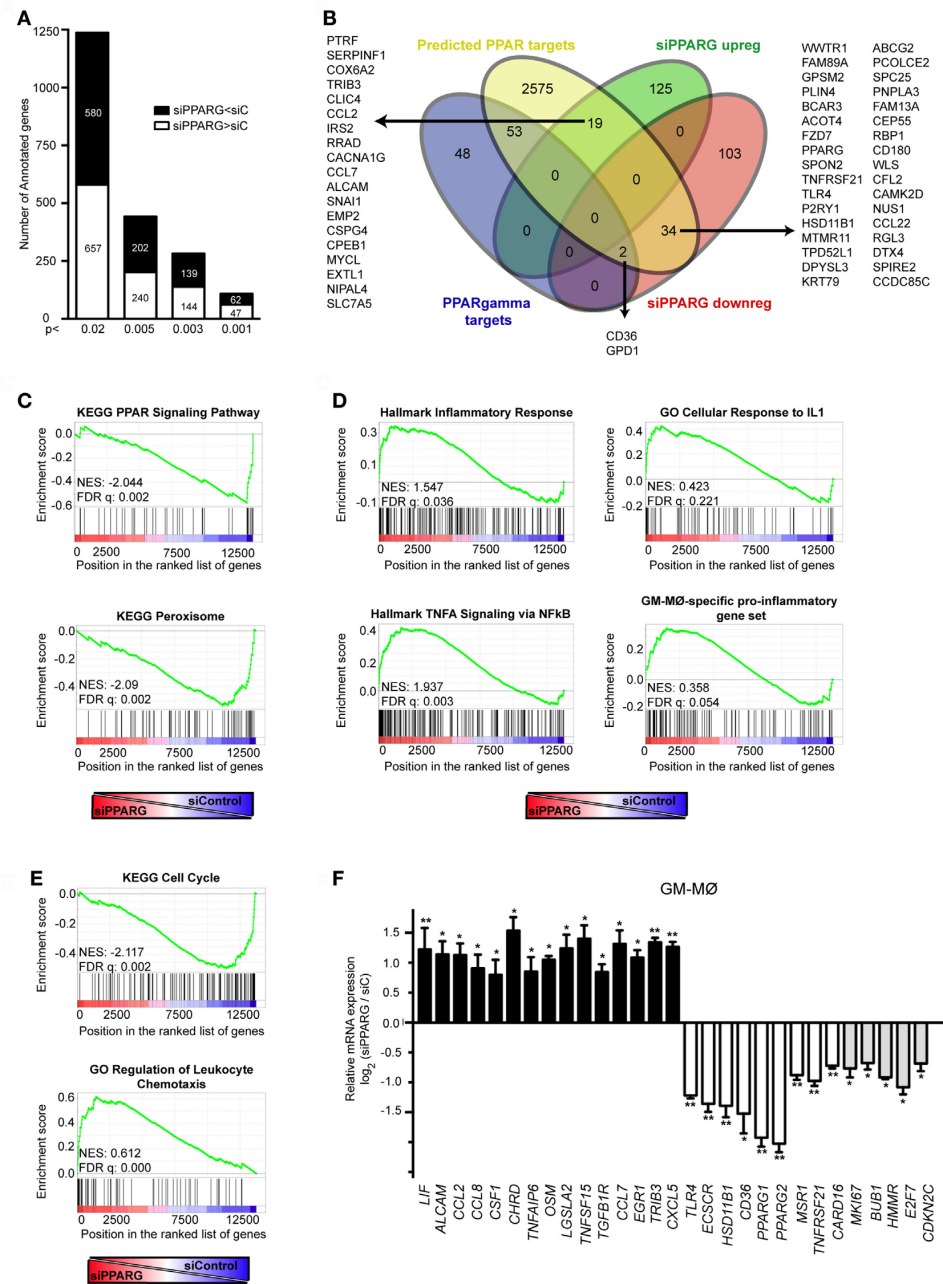
Peroxisome proliferator-activated receptor gamma (PPARγ) controls the global transcriptional signature of GM-CSF-conditioned human monocyte-derived macrophages (GM-MØ). **(A)** Number of annotated genes whose expression is higher or lower in siPPARG-transfected than in siControl-transfected (*siC*) GM-MØ at the indicated *p*-values. **(B)** Venn diagram analysis of the genes differentially expressed in siPPARG-transfected and siControl-transfected GM-MØ compared to experimentally verified PPARγ target genes (PPARγ targets) and computationally predicted PPAR target genes (predicted PPAR targets), as reported in the PPARgene database (53). **(C–E)** Gene set enrichment analysis on the “*t* statistic-ranked” list of genes obtained from the siPPARG-GM-MØ versus siControl-GM-MØ limma analysis, using the indicated gene set. In **(D)**, the previously defined GM-MØ-specific “*Proinflammatory gene set*” (12) was also used. **(F)** Expression of the indicated genes in siPPARG-transfected and siControl-transfected (*siC*) GM-MØ, as determined by quantitative real-time PCR on three to five independent GM-MØ samples. Results are indicated as the mRNA levels of each gene in siPPARG-transfected relative to the levels in siControl-transfected GM-MØ (*n* = 3–5; *p < 0.05; **p < 0.01).

Given the transcriptional effects of PPARγ knockdown (Figure 1C), and to more thoroughly address the role of PPARγ in *in vitro* generated GM-CSF-conditioned macrophages, we determined the PPARγ-dependent transcriptional profile of GM-MØ in the absence of exogenous agonists. siRNA-mediated PPARγ knockdown significantly modified the transcriptome of GM-MØ, altering the expression of 314 probes (283 annotated genes) (*p* < 0.003, Table S2 in Supplementary Material). Specifically, PPARγ knockdown led to downregulation of 139 genes and upregulation of 144 genes in GM-MØ (Figure 5A). Twenty-five percent of the genes downregulated by siPPARG (36 out of 139) had been previously predicted as PPAR targets (53), including 20 genes upregulated by long-term rosiglitazone treatment of human monocyte-derived dendritic cells (54) and two genes whose expression is also diminished in mouse Pparγ^−/−^ macrophages (*CD36* and *GPD1*) (24) (Figure 5B). Similarly, the set of genes upregulated upon PPARγ knockdown contained 19 genes predicted as PPAR targets (53) (Figure 5B), including 5 genes upregulated by rosiglitazone in human dendritic cells (54) and *CCL2* and *CCL7*, whose orthologous genes are overexpressed in murine Pparγ^−/−^ macrophages (24). Conversely, siPPARG downregulated the expression of *MSR1*, whose mouse ortholog is overexpressed in Pparγ^−/−^ macrophages (24). The PPARγ-regulated gene set also included genes whose expression distinguishes A-MØ from other tissue-resident mouse macrophages (*KRT79, BCAR3, MAFF, WWTR1*) (55) or have been defined as human A-MØ-enriched genes (*EDN1, CXCL1, TNFAIP6, IL7R*) (56) (Table S2 in Supplementary Material). Therefore, PPARγ knockdown in GM-CSF-conditioned human macrophages allowed the identification of a large set of genes (Table S2 in Supplementary Material) whose expression is specifically modulated by PPARγ in the absence of an exogenous agonist. Besides, and in agreement with the divergent transcriptional profiles of functionally similar human and mouse macrophages (57), the human macrophage PPARγ-dependent gene set in human macrophages only partially overlaps with the list PPARγ-regulated genes previously identified in mouse macrophages.

To gain formation on the biological processes significantly affected after PPARγ knockdown in human macrophages, functional enrichment analysis was performed using GSEA (50). Confirming the validity of the results, PPARγ knockdown led to a very significant reduction in the expression of genes associated with the terms “KEGG_PPAR_Signaling_Pathway” and “KEGG_Peroxisome” (Figure 5C). In line with its known anti-inflammatory function (32, 58), reduction of PPARγ expression in GM-MØ caused a significant increase in the expression of genes within the “Hallmark_Inflammatory Response,” “Hallmark_TNFA signaling *via* NFKB,” and “GO_Cellular Response to IL1” gene sets (Figure 5D). Also in agreement with the anti-inflammatory activity of PPARγ (32, 58), PPARγ knockdown promoted a significant global upregulation of the GM-MØ-specific “*Proinflammatory gene set*” (12) (Figure 5D), and specially of two GM-MØ-specific genes like *ECSCR* and *HSD11B1* (11) (Table S2 in Supplementary Material). Unexpectedly, PPARγ knockdown resulted in a very significant increase in the expression of genes within gene sets directly involved cell cycle and proliferation like “KEGG Cell Cycle,” “Reactome Mitotic M G1 Phases,” “Reactome G1 S Transition,” “Hallmark G2M Checkpoint,” “GO Cell Division,” and others (all with FDR *q* = 0.000) (Figure 5E and not shown). Besides, reduction of PPARγ caused a very significant increase in the expression of genes involved in chemotaxis (Figure 5D), suggesting that PPARγ has a negative regulatory effect on leukocyte mobility. All these results were further supported after ENRICHR analysis (see text footnote 4) (48, 49), which also revealed that PPARγ knockdown specifically impairs the expression of genes whose expression is controlled by a known PPARγ ligand (rosiglitazone, adjusted *p* = 1.4 × 10^−10^) and a transcription factor (FOXM1, adjusted *p* = 8.47 × 10^−18^) that collaborates with PPARγ in pulmonary inflammation (59, 60) (Table S2 in Supplementary Material and data not shown).

To further validate the correlations found with GSEA and ENRICHR, a representative number of transcriptional changes were assessed after PPARγ knockdown on independent GM-MØ preparations (Figure 5F). PPARγ knockdown significantly reduced the expression of genes encoding PAMP/DAMP receptors (*TLR4, CD36*), NFκB activators (*TNFRSF21, CARD16*), the scavenger receptors SR-A1 (*MSR1*), known PPARγ targets like *HSD11B1*, and the GM-MØ-specific gene ECSCR (11) (Figure 5F). Conversely, reduction of PPARγ levels increased the expression of genes encoding cytokines (*CCL2, CCL7, CCL8, CXCL5*), again pointing toward a negative regulatory effect of PPARγ on the expression of genes associated with cell chemotaxis. PPARγ knockdown also enhanced expression of genes encoding various growth-promoting factors (*CSF1, TNFSF15, OSM, LIF)* (Figure 5F). Moreover, downregulating PPARγ expression led to a significant reduction in the expression of genes that contribute to the significant enrichment signal (“leading-edge”) found after GSEA on gene sets related to cell cycle and proliferation (namely *MKI67, BUB1, HMMR, E2F7*, and *CDKN2C*) (Figure 5F, gray-filled bars) and that code for proteins involved in cell cycle regulation like Ki67, E2F7, and CDKN2C. Therefore, and in agreement with the GSEA correlations (Figure 5E), expression of PPARγ in GM-CSF-conditioned macrophages has a positive impact on the expression of genes that directly regulate and mark cell proliferation.

### PPARγ-Dependent Gene Profile in Human A-MØ

**Figure 6:**
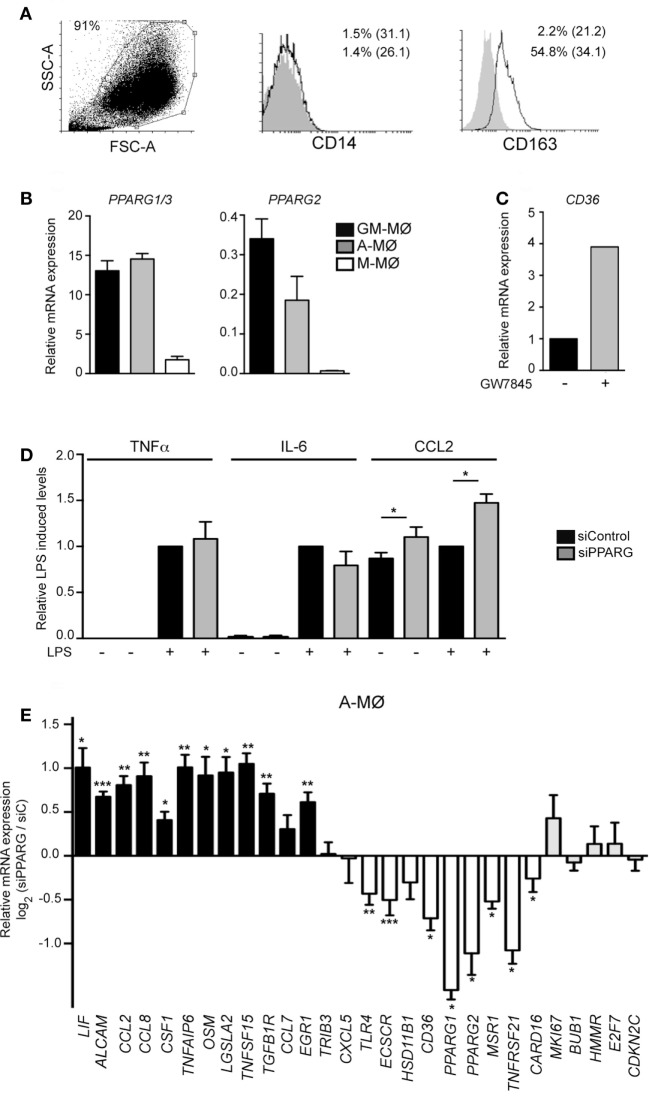
Peroxisome proliferator-activated receptor gamma (PPARγ) controls the transcriptome of human alveolar macrophages. **(A)** Cell surface expression of CD14 and CD163 in Alveolar macrophage (A-MØ) isolated from a representative bronchoalveolar lavage, as determined by flow cytometry. Background fluorescence was determined using isotype-matched antibodies (gray histograms). Forward scatter (FSC)/Side scatter (SSC) analysis of isolated A-MØ is shown in the left panel. **(B)** *PPARG1/3* and *PPARG2* mRNA expression in siControl-transfected A-MØ, GM-MØ, and M-CSF-dependent monocyte-derived macrophages (M-MØ), as determined by quantitative real-time PCR (qRT-PCR) and relative to *TBP* mRNA levels. Mean and SEM of three independent experiments is shown. **(C)** *CD36* mRNA expression in A-MØ cultured for 24 h after isolation in the presence of GW7845 (1 µM) or DMSO (−), as determined by qRT-PCR. Results are indicated relative to the *CD36* mRNA expression in the presence of DMSO (arbitrarily set to 1). One representative experiment is shown. **(D)** TNFα, IL-6, and CCL2 production in LPS-treated (24 h) siPPARG-transfected and siControl-transfected A-MØ. Results indicate the concentration of each cytokine for each condition relative to the cytokine levels detected in siControl-transfected A-MØ (arbitrarily set to 1). Mean and SEM of three independent experiments are shown (*p < 0.05). **(E)** Expression of the indicated genes in siPPARG-transfected and siControl-transfected (*siC*) A-MØ, as determined by qRT-PCR. Results are indicated as the mRNA levels of each gene in siPPARG-transfected relative to the levels in siControl-tranfected (*siC*) A-MØ (*n* = 6–7; *p < 0.05; **p < 0.01; ***p < 0.001).

To assess the physiological relevance of the above findings, we next analyzed the functional and transcriptional consequences of siRNA-mediated PPARγ knockdown in human A-MØ isolated from BAL fluids. In agreement with previous reports (61, 62), isolated A-MØ expressed CD163 and exhibited very low levels of cell surface CD14 (Figure 6A). Regarding PPARγ expression, A-MØ exhibited a level of *PPARG1/3 and PPARG2* mRNA expression similar to that found in GM-MØ and considerably higher than the expression seen in M-MØ (Figure 6B), and responded to the presence of the PPARγ agonists GW7845 by enhancing the expression of *CD36* (Figure 6C). At the functional level, PPARγ knockdown in A-MØ did not significantly modify the LPS-induced production of TNFα or IL-6 (Figure 6D), thus indicating that PPARγ does not regulate the LPS-induced production of proinflammatory cytokines by A-MØ in the absence of an exogenous agonist, a result also seen in monocyte-derived GM-MØ (Figure 1G). By contrast, PPARγ knockdown significantly enhanced CCL2 production by human A-MØ both under basal and LPS-stimulated conditions (Figure 6D). Regarding the transcriptional role of PPARγ in *ex vivo* isolated human A-MØ, PPARγ silencing in A-MØ yielded similar effects to those previously observed in GM-MØ (Figure 6E). Specifically, PPARγ knockdown caused a significant upregulation of *CCL2, CCL8, CSF1, TNFSF15, OSM*, and *LIF*, and a significant downregulation of *TLR4, ECSCR, CD36, MSR1, TNFRSF21*, and *CARD16* (Figure 6E). However, and in contrast with its effects on GM-MØ, downregulation of PPARγ in human A-MØ had no effect on the expression of genes encoding proteins involved in cell cycle regulation (Figure 6E). Altogether, these results indicate that PPARγ significantly contributes to the transcriptional signature of GM-CSF-conditioned human macrophages (either proinflammatory monocyte-derived GM-MØ or *ex vivo* isolated A-MØ) in the absence of exogenous agonists.

## Discussion

Macrophages adapt to changes in the extracellular environment very efficiently and alter their phenotype and effector functions according to their surrounding milieu. Although GM-CSF-conditioned monocyte-derived macrophages (GM-MØ) produce large amounts of proinflammatory cytokines upon TLR stimulation, they express high levels of PPARγ, whose ligand-induced activation down-modulates macrophage proinflammatory responses (35, 36), skews monocytes toward an anti-inflammatory phenotype (38, 63, 64), and limits inflammation in metabolically activated macrophages (39). Assessment of the function of PPARγ in human macrophages has now revealed that (1) the range of PPARγ target genes differs between proinflammatory (GM-MØ) and anti-inflammatory (M-MØ) monocyte-derived macrophages; (2) *PPARG1/3* and *PPARG2* are preferentially expressed by human GM-MØ; (3) activin A mediates the GM-CSF-induced expression of PPARγ in *in vitro* generated GM-MØ and *ex vivo* isolated A-MØ; and (4) PPARγ shapes the gene signature of GM-CSF-conditioned human macrophages in the absence of exogenous agonists. The involvement of activin A in PPARγ expression in GM-CSF-dependent human macrophages points toward a connection between Smad2/3 activation and *PPARG* gene expression, whose existence has been already suggested in PAP patients, which exhibit a deficiency in PPARγ and a severe reduction in Activin A expression and secretion (42). Thus, our results provide a molecular explanation for such a correlation, and support the existence of a functional GM-CSF/activin A/PPARγ axis in human macrophages.

The higher PPARγ expression exhibited by human proinflammatory GM-MØ is reminiscent of the differential PPARγ levels seen in mouse Ly-6C^hi^ (low PPARγ) and peripheral blood monocytes (34). From this point of view, and since PPARγ^high^ Ly-6C^lo^ monocytes are thought to become “M2 polarized” macrophages within tissues (and PPARγ^low^ Ly-6C^hi^ monocytes are thought to give rise to “M1 polarized macrophages”), the expression of PPARγ might mark macrophages with distinct inflammatory activities in mice and humans. A possible explanation for this discrepancy could derive from the fact that PPARγ^low^ Ly-6C^hi^ monocytes are precursors of PPARγ^high^ Ly-6C^lo^ monocytes (65), and that exposure of M-MØ (low PPARγ) to GM-CSF leads to enhanced expression of PPARγ. Therefore, it is tempting to speculate that high PPARγ expression marks macrophages (murine and human) that have been already exposed to an activating/proinflammatory stimulus. Moreover, the preferential expression of *PPARG1* and *PPARG2* in proinflammatory GM-MØ is in apparent contradiction with the correlation between PPARγ expression and the presence of M2/anti-inflammatory markers in macrophages from human carotid atherosclerotic lesions (63), and with the contribution of PPARγ to the IL-4- and STAT6-dependent M2 macrophage polarization (38). The higher levels of *PPARG* seen in GM-MØ might be related to the acquisition of the ability to halt proinflammatory responses in a fast and efficient manner, allowing macrophages to rapidly switch from a proinflammatory into an anti-inflammatory polarization state to avoid excessive tissue damage before restoring homeostasis. This explanation is compatible with the preferential expression of other anti-inflammatory/immunosuppressive genes like *VDR* or *HSD11B1* (11) in GM-MØ (GSE27792) and with the ability of PPARγ to limit inflammation in macrophages metabolically activated by glucose, insulin and palmitate (39).

Apart from the polarization-dependent expression of PPARγ (Figure 3) and the distinct cytokine responsiveness of *PPARG* expression in GM-MØ and M-MØ (data not shown), we have found that the range of genes specifically modulated by the PPARγ agonist GW7845 differs between proinflammatory GM-MØ and anti-inflammatory M-MØ (Figure 1). Although some of the differential PPARγ target genes had been shown to be modulated by PPARγ in various cell types (66–70), the distinct transcriptional consequence of PPARγ activation in human GM-MØ or M-MØ was, to our knowledge, so far unknown. Importantly, PPARγ activation also has different functional consequences in both macrophage subtypes, because the PPARγ agonist GW7845 significantly inhibit the LPS-induced production of proinflammatory cytokines (IL-6, TNFα) in GM-MØ, but has no effect on M-MØ. Therefore, our results indicate that PPARγ activation leads to distinct outcomes in human macrophages exhibiting opposite transcriptional and functional profiles (GM-MØ and M-MØ). This result agrees with those reported by Bouhlel et al. (63), who found that PPARγ activation exclusively modulates CD163 and CD206 in IL-4-polarized macrophages, and supports the polarization-dependent activity of PPARγ in macrophages. Our results on human macrophages are also in line with the divergent PPARγ binding landscape reported in human and mouse macrophages (71), as *CCL2* and *IL10* mRNA levels, exclusively downregulated by GW7845 in GM-MØ, were diminished in both murine macrophage subtypes upon PPARγ activation, whereas *THBS1* mRNA, whose levels were increased in human GM-MØ, were diminished in murine GM-MØ in response to GW7845.

The definition of the PPARγ-dependent transcriptome in GM-MØ also provides evidences to support that PPARγ is transcriptionally active in human macrophages not exposed to exogenous pharmacological PPARγ agonists. The relevance of this finding is further reinforced by the effect of PPARγ knockdown on the gene profile of *ex vivo* isolated A-MØ. Therefore, down-modulation of PPARγ expression suffices to alter the transcriptome of GM-CSF-conditioned human macrophages (*in vitro* generated GM-MØ and *ex vivo* isolated A-MØ) but does not influence their LPS-induced proinflammatory cytokine production. This feature suggests that PPARγ has a distinct role in resting and activated (e.g., LPS-exposed) macrophages. In the former, PPARγ shapes the macrophage transcriptome, positively regulating genes that encode Th2 cytokine-induced chemokines (*CCL13, CCL22*) and *TLR4*, and downregulating genes that code for NFκB-regulated monocyte-attracting chemokines (*CCL2, CCL8*). Both consequences are compatible with the function of PPARγ^+^ A-MØ, that remove airborne particles and pathogens while avoiding lung inflammatory responses, and indicate a prominent role for the PPARγ transcriptional activating ability in non-activated human macrophages. Conversely, the ability of PPARγ to impair proinflammatory cytokine production after LPS stimulation is only observed after agonist-induced activation, thus suggesting that the anti-inflammatory ability of PPARγ is displayed in full only upon macrophage activation.

In summary, we report that the functions of PPARγ in human macrophages are polarization-dependent, that activin A positively regulates PPARγ expression in GM-CSF-dependent macrophages, and that PPARγ shapes the transcriptome of GM-CSF-conditioned human macrophages in the absence of exogenous agonists. Regarding the latter, the large set of potential novel PPARγ target genes now identified in human macrophages, which code for molecules involved in PAMP and DAMP recognition, inflammatory cell migration, proliferation promotion and cell cycle progression, is indicative of the role of PPARγ in regulation of inflammatory responses and in defense against pathogens, and further supports its contribution to maintenance of lung homeostasis.

## Ethics Statement

Buffy coats were obtained from healthy blood donors, as anonymously provided by the Comunidad de Madrid blood Bank. Ethical approvals for all blood sources and processes used in this study were approved by the Centro de Investigaciones Biológicas Ethics Committee. All experiments were carried out in accordance with the approved guidelines and regulations. All experiments on mice were conducted according to the Spanish and European regulations on care and protection of laboratory animals and were approved by the Centro de Investigaciones Biológicas animal facility and the Consejo Superior de Investigaciones Científicas Ethics Committee.

## Author Contributions

CN, RB, CM, ES-F, BA, ME, JD-A and CA designed research, performed research, and analyzed data; AC and MAV designed research and analyzed data; CN, AP-K, and ALC conceived the study, designed research, analyzed data, and wrote the article. All authors had final approval of the version.

## Conflict of Interest Statement

The authors declare that the research was conducted in the absence of any commercial or financial relationships that could be construed as a potential conflict of interest.

## Footnotes

^1^http://www.bioconductor.org

^2^http://www.r-project.org

^3^http://www.ncbi.nlm.nih.gov/geo

^4^http://amp.pharm.mssm.edu/Enrichr/
