# Supplementary material for: The Activin A-Peroxisome Proliferator-Activated Receptor Gamma Axis Contributes to the Transcriptome of GM-CSF-Conditioned Human Macrophages
Source: Front Immunol. 2018 Jan 29;9:31. doi: 10.3389/fimmu.2018.00031 (PMC5796898; doi:10.3389/fimmu.2018.00031)
Supplement: Supplementary file 1 [file Table_1.PDF]

SUPPLEMENTARY TABLE I. List of oligonucleotides used in this study.

HUMAN

GENE	OLIGO SENSE	OLIGO ANTISENSE
PPARG1	gacaggaaagacaacagacaaatc	ggggtgatgtgtttgaacttg
PPARG2	tccatgctgttatgggtgaa	tgtgtcaaccatggtcatttc
FLT1	cagcatacctcactgttcaagg	ccacacaggtgcatgttagag
IL6	caggagcccagctatgaact	gaaggcagcaggcaacac
THBS1	caatgccacagttcctgatg	tggagaccagccatcgtc
CD36	tggaacagaggctgacaactt	ttgattttgatagatatgggatgc
CCR2	tgagacaagccacaagctga	ttctgataaaccgagaacgag at
IL10	tcactcatggctttgtagatgc	gtggagcaggtgaagaatgc
CCL2	agtctctgccgcccttct	gtgacggggcattgattg
CSF1	gcaagaactgcaacaacagc	agttcgaatcaggcttggtc
HAMP	agacagac gcacgatgg	gcagctctgcaagttgtcc
FABP4	cctttaaaaatactgagatttccttca	ggacacccccatctaaggtt
HMMR	gatgttactgctcaatttgaaagc	gccgctttttcctgtaatga
MSR1	tttgatgctcgctcaatgac	ttgaagggaagggctgtttt
E2F7	cagttcaggcttctgagagga	ttctaaagagtagccacctgatcc
CARD16	gccaaatttgcatcacatacat	gtcctgcactgcctgaaga
TNFRSF21	gcacatggaaacccatgaa	agaagagttggattctgttgagttc
CDKN2C	agagatctgtagcgtaggtacgtg	acatacattcctggttaatgactcc
BUB1	tgcatttgaagcccagttt	caaagaagagatgatcttattgactcc
CHRD	ccagccaggaggacacac	gtgcccacgttcaggaag
TNFSF15	caagggcacacctgacagt	cctagttcatgttcccagtgc
LGALS2	ggttaagaacatggacatgaagc	tggcccagattaattacaaagc
ALCAM	caggttcctgccgtctgct	ctgaatttacagtataccatccaagg
LIF	tgccaatgccctctttattc	gtccaggttgttggggaac
OSM	acagaggacgctgctcagtc	ggtgtcctgcatgagatctgt
CCL7	ggcttgctcagccagttg	cctgccctgcacagttaca
CXCL5	ggtccttcgagctccttgt	acgcagctctctcaacacag
TNFAIP6	ggccatctcgcaacttaca	cagcacagacatgaaatccaa
TRIB3	gtcttcgctgaccgtgaga	cagtcagcacgcaggagtc
EGR1	agccctacgagcacctgac	ggtttggctggggtaactg
TGFBR1	aaattgctcgacgatgttcc	cataataaggcagttggtaatcttca
CCL8	ccctcagggacttgctcag	ctccagcctctggataggaa
TLR4	cctgcgtgagaccagaaag	ttcagctccatgcattgataa
ECSCR	agctgtgctgggtgatcct	attgtgggctgggagttg t
HSD11B1	caatggaagcattgttgtcg	ggcagcaaccattggataag
ABCA1	tgctgcatagtcttgggactc	acctcctgtcgcatgtcact
TBP	cggctgtttaacttcgcttc	cacacgccaagaaacagtga
GAPDH	agccacatcgctcagacac	gcc aatacgaccaaatcc
HPRT1	tgaccttgatttattttgcatacc	cgagcaagacgttcagtcct

MOUSE

GENE	OLIGO SENSE	OLIGO ANTISENSE
Pparg1	gaaagacaacggcaaatcacc	gggggtgatatgtttgaacttg

Pparg2	tgctgttatgggtgaaactctg	ctgtgtcaaccatggtaatttctt
Cd36	ttgtacctatactgtggctaaatgaga	cttgtgttttgaacatttctgctt
Ccr2	acctgtaaatgccatgcaagt	tgtcttccatttcctttgatttg
Csf1	caacagctttgctaagtgctcta	cactgctaggggtggcttta
Thbs1	cacctctccgggttactgag	gcaacaggaacaggacaccta
Ccl2	catccacgtgttggctca	gatcatcttgctggtgaatgagt
Hamp	gatggcactcagcactcg	ctgcagctctgtagtctgtctca
Il10	cagagccacatgctccta	gtccagctggtcctttgt
Flt1	ggcccgggatatttataagaac	ccatccattttaggggaagtc
Tbp	ggggagctgtgatgtgaagt	ccaggaaataattctggctca
gapdh	actgtggatggcccctctgg	tgaccttgcccacagccttg
