# Supplementary material for: The Activin A-Peroxisome Proliferator-Activated Receptor Gamma Axis Contributes to the Transcriptome of GM-CSF-Conditioned Human Macrophages
Source: Front Immunol. 2018 Jan 29;9:31. doi: 10.3389/fimmu.2018.00031 (PMC5796898; doi:10.3389/fimmu.2018.00031)
Supplement: Supplementary file 2 [file Table_2.PDF]

Supplementary Table II

List of genes significantly (p<0.003) altered after PPARγ knockdown in GM-MØ

(only genes with fluorescence expression >100 in GM-MØ transfected with siControl or siPPARγ are shown)

Gene symbol	log2 fold	p	Fluorescence level			Gene symbol	log2 fold	p	Fluorescence level	
			siControl	siPPARγ					siControl	siPPARγ
GPD1	-1.67	0	1286.24	421.39		*NIPAL4*	1.4267	2.00E-04	292.53	817.89
PTPRF	-1.5067	0	296.74	104.94		*LOC101927440*	1.24	7.00E-04	413.52	1023.65
NAGA	-1.4433	0	398.03	146.04		*lnc-C11orf44-2*	1.1967	0.0021	103.58	242.4
GOLGA7B	-1.3967	2.00E-04	726.95	264.3		*LOC283485*	1.17	2.00E-04	180.79	400.89
PPARG	-1.3633	2.00E-04	3096.1	1271.76		*PXYLP1*	1.16	0.0015	218.15	479.54
TPD52L1	-1.3567	1.00E-04	442.42	178.84		*WIPF3*	1.15	0.0012	172.63	355.19
PCOLCE2	-1.3133	6.00E-04	1321.04	626.03		*STEAP3*	1.1067	4.00E-04	1093.39	2382.62
CCL22	-1.3067	6.00E-04	8018.05	3279.17		*S100A5*	1.08	4.00E-04	579.95	1258.41
SPOCD1	-1.2567	3.00E-04	18156.6	7629.8		*LRRC7*	1.0533	7.00E-04	134.8	291.5
PPARG	-1.23	0	958.78	411.02		*HOPX*	1.05	0.0023	288.19	613.75
CCDC85C	-1.1767	1.00E-04	303.38	130.43		*lnc-ZNF236-1*	1.0433	1.00E-04	570.44	1177.21
PNPLA3	-1.1567	0	3274.17	1475.83		*lnc-FLYWCH2-1*	1	0.0024	558	1157.92
CCDC162P	-1.12	8.00E-04	795.43	358.86		*CYP19A1*	0.9833	8.00E-04	500.29	937.23
ME3	-1.1067	1.00E-04	1705.71	786.24		*CSPG4*	0.9833	5.00E-04	170.22	324.06
PHACTR1	-1.0767	3.00E-04	428.65	214.06		*PROC*	0.98	2.00E-04	488.84	954.23
PODXL	-1.0733	3.00E-04	4470.29	2283.45		*SLC7A5*	0.9767	5.00E-04	242.49	456.11
MS4A14	-1.07	2.00E-04	483.16	235.84		*CHRD*	0.9733	2.00E-04	203.26	399.46
C2orf71	-1.0333	0.0015	390.76	189.51		*SCN3A*	0.9733	9.00E-04	140.47	282.58
FAM13A	-1.0233	0.0011	356.31	174.86		*TNFSF15*	0.9533	3.00E-04	174.98	334.29
MS4A7	-1.02	1.00E-04	11194.59	5552.5		*OR2M7*	0.9533	0.0026	125.24	249.4
GPA33	-1.0033	3.00E-04	446.81	238.27		*TRIB3*	0.9067	0.0018	3212.32	6443.59
FAM89A	-0.9967	4.00E-04	2409.91	1270.43		*PROC*	0.89	4.00E-04	2364.66	4314.92
MOGAT1	-0.9833	3.00E-04	214.69	109.2		*ARHGEF10*	0.8833	4.00E-04	189.09	358.89
IL7R	-0.9567	0.0014	1354.15	715.54		*lnc-RLBP1-1*	0.8733	0.0011	230.86	424.99
CCL13	-0.9567	0.0029	416.47	184.54		*lnc-ZAP70-2*	0.87	5.00E-04	344.96	647.45
AMDHD1	-0.9467	2.00E-04	1010.18	522.72		*PTRF*	0.8667	0.001	3211.03	5646.33
LOC731424	-0.9467	0.0015	246.38	129.69		*LGALS2*	0.8633	0.0023	31714.88	52724.51
S100A16	-0.9433	4.00E-04	725.74	386.83		*ALCAM*	0.8567	4.00E-04	4546.22	8080.47
CD36	-0.94	2.00E-04	7910.18	4213.28		*LIF*	0.8567	0.0015	151.99	262.06
NUPL1	-0.92	2.00E-04	842.64	443.43		*TLN1*	0.8533	5.00E-04	1131.11	2033.95
DUSP13	-0.9033	4.00E-04	4344.86	2286.27		*LOC100129455*	0.8533	0.0025	196.25	354.73
CHI3L2	-0.8867	0.0013	585.44	324.55		*CCL7*	0.85	0.0021	5662.58	10127.66
WWTR1	-0.88	2.00E-04	1356.26	735.86		*CHPF*	0.8467	7.00E-04	2068.21	3735.93
S100P	-0.86	5.00E-04	912.63	500.15		*OSM*	0.83	0.0015	698.32	1224.49
MGAT3	-0.8467	0.001	802.61	428.27		*EMP2*	0.83	0.001	218.15	407.13
RBP1	-0.8333	0.0027	270.37	152.68		*C1orf140*	0.8167	8.00E-04	345.99	618.61
ECSCR	-0.83	0.0011	585.79	329.38		*CXCL5*	0.8133	6.00E-04	11183.07	19147.59
SPIRE2	-0.8267	9.00E-04	415.02	244.28		*ISYNA1*	0.8133	7.00E-04	1143.81	2051.24
ADTRP	-0.81	3.00E-04	790.08	449.63		*POT1*	0.8067	0.0014	2309.34	4138.66
TEX14	-0.8067	0.0014	242.81	136.38		*POLR3E*	0.8033	0.0011	532.36	926.38
HSD11B1	-0.8	8.00E-04	4791.71	2831.94		*lnc-SPAG1-4*	0.8033	7.00E-04	241.58	418.37
DYX1C1	-0.8	3.00E-04	677.66	389.69		*ISYNA1*	0.79	5.00E-04	635.82	1114.28
TOP2A	-0.7967	9.00E-04	200.92	114.98		*GNB1*	0.7833	0.0013	5280.33	9025.71
GGTA1P	-0.78	0.0015	4666.72	2691.51		*TBXA2R*	0.7767	5.00E-04	684.88	1162.85
TLR4	-0.7733	7.00E-04	1010.41	596.66		*CSGALNACT1*	0.7767	0.0014	564.84	928.79
KCNK13	-0.77	9.00E-04	299.9	173.86		*DACT3*	0.7733	8.00E-04	249.25	424.15
MAFF	-0.7667	6.00E-04	2363.34	1400.03		*AANAT*	0.7533	5.00E-04	228.81	393.05
CENPA	-0.7633	0.0021	189.4	108.84		*lnc-CCDC69-1*	0.7433	0.0011	311.54	529.23
DPYSL3	-0.76	4.00E-04	501.27	292.87		*ATP2B2*	0.7333	6.00E-04	214.71	359.82
HMMR	-0.7533	7.00E-04	1865.33	1080.39		*STEAP1*	0.73	0.0023	100.21	161.47
IKZF2	-0.7533	4.00E-04	850.04	508.63		*AGO4*	0.7233	0.0026	413.33	690.09
NMB	-0.75	4.00E-04	14921.37	8895.74		*LINC00877*	0.7233	0.0015	300.14	507.7
NAGA	-0.7433	0.001	7036.14	4147.48		*ASB2*	0.72	5.00E-04	1756.65	2890.42
DDIAS	-0.7433	0.0015	1321.29	784.19		*TNFAIP6*	0.7167	0.0022	621.66	938.29
SVIL	-0.74	7.00E-04	388.74	232.37		*IRS2*	0.7133	0.0013	1067.91	1738.29
MSR1	-0.7333	7.00E-04	4050.67	2448.74		*TUBA8*	0.7067	0.0015	1837.49	2876.47
GPR34	-0.7333	7.00E-04	2752.56	1692.11		*CREM*	0.6967	0.0015	748.12	1211.48
DTX4	-0.7067	9.00E-04	287.01	173.22		*MAGEA6*	0.6967	0.0025	278.66	464.16
MSRB1	-0.7	8.00E-04	7982.12	4928.12		*PRSS30P*	0.6967	0.0016	283.84	447.9
HOXB6	-0.6967	8.00E-04	241.71	145.69		*lnc-TSPYL6-2*	0.69	0.0021	232.52	381.89
NUS1	-0.69	0.001	8180.14	5064.24		*ELOVL7*	0.69	6.00E-04	128.43	207.38
KRT79	-0.69	7.00E-04	549.89	342.92		*CCL2*	0.6867	0.0029	20701.07	33129.03
SIRPG	-0.69	0.0013	531.29	331.49		*DRAM1*	0.6767	9.00E-04	6806.72	10881.01
BCAR3	-0.6867	7.00E-04	708.6	441.13		*CLIC4*	0.6767	0.001	1722.32	2731.83
CEP55	-0.6833	0.0016	1176.26	721.59		*EGR1*	0.6733	8.00E-04	366.62	583.42
ID4	-0.6833	8.00E-04	305.48	190.36		*CSPG5*	0.6733	0.0018	275	447.46
E2F7	-0.68	0.0022	242.66	147.49		*SERPINF1*	0.6733	0.0017	227.27	361.61
SEL1L2	-0.68	0.0011	227.06	140.88		*CHST2*	0.67	9.00E-04	398	628.9
CARD16	-0.6733	9.00E-04	2574.2	1616.89		*CPEB1*	0.6667	0.0026	968.48	1565.29
CKAP2L	-0.67	0.0014	162.48	100.92		*LINC01478*	0.6667	0.0026	166.22	270.01
AKR1B1	-0.6667	0.0017	7015.51	4367.91		*lnc-SBDS-10*	0.6667	0.0015	118.48	188.98
TLR4	-0.6667	8.00E-04	3540.52	2234.11		*SMIM1*	0.6633	0.0016	1024.82	1618.31
C20orf194	-0.6567	0.0012	2798.11	1767.74		*lnc-CTNNA2-1*	0.6567	0.0021	4541.54	7281.1
ACOT4	-0.6367	0.0012	2299.94	1491.24		*SLMO1*	0.6567	0.0017	847.7	1325.73
PLIN4	-0.6367	0.0023	847.6	546.36		*NLRP12*	0.6567	0.0028	332.38	525.78
S100A9	-0.6333	0.0014	15945.95	10154.97		*TMEM132A*	0.65	0.0028	735.39	1107.39
LOC644189	-0.6333	0.0027	3518.25	2259.62		*BPI*	0.64	0.0011	282.54	447.43
EPB41L1	-0.6267	0.0022	11605.51	7584.55		*LOC284379*	0.6333	0.0025	106.96	166.41
PPP2R3A	-0.6167	0.0016	2336.37	1517.76		*lnc-KIAA1210-2*	0.63	0.0014	171.85	268.33
TM4SF1	-0.6133	0.0013	833.61	543.79		*TGFBR1*	0.6267	0.0021	561.84	869.42
RAB42	-0.6067	0.0012	4315.57	2828.73		*C16orf89*	0.6133	0.0027	323.35	495.98
TNFRSF21	-0.6	0.0023	10078.47	6646.56		*DMKN*	0.6033	0.0013	220.59	335.01
EMR3	-0.5967	0.0017	258.89	177.68		*NT5M*	0.5967	0.0021	1383.18	2081.94
NEU1	-0.5933	0.0015	10298.61	6827.71		*ST7L*	0.5967	0.0026	186.98	283.06
DNAJC6	-0.5933	0.0026	323.12	213.41		*lnc-CCDC107-2*	0.5967	0.0013	137.36	207.73
lnc-ATF3-1	-0.59	0.0015	229.68	155.13		*ANKRD46*	0.5933	0.0024	182.01	273.14
NUSAP1	-0.5833	0.0018	1408.79	933.13		*FSCN2*	0.5933	0.0027	155.21	238.49

KIF14	-0.5833	0.0027	326.54	215.07		***LOC100506085***	0.59	0.0025	197.31	297.28
SPC25	-0.5767	0.0028	630.86	416.9		***SPOPL***	0.5867	0.0021	316.04	472.45
ESCO2	-0.57	0.0021	155.83	104.69		***CXCL1***	0.58	0.0024	711.25	1056.13
ASPM	-0.5667	0.0025	1322.79	879.62		***APCS***	0.5667	0.0021	135.11	200.41
DEPDC1	-0.5633	0.0026	740.27	496.67		***MYCL***	0.5633	0.0021	1816.53	2681.83
MSR1	-0.56	0.0019	8709.27	5914.93		***CCL8***	0.5633	0.0023	139.52	203.49
KAL1	-0.5567	0.0025	252.71	176		***ZBTB47***	0.5567	0.0027	2093.03	3059.21
DNMT3B	-0.5567	0.0029	200.42	135.04		***lnc-TEFM-2***	0.5567	0.0021	1010.11	1486.48
PLA2G15	-0.5533	0.0019	73943.01	50365.44		***LINC00323***	0.5467	0.0027	268.47	390.13
lnc-RP11-150O12	-0.5533	0.0029	298.52	201.74		***TMEM235***	0.5467	0.0021	117.12	172.58
SLC19A2	-0.55	0.0024	556.56	379.61		***ATP2A3***	0.5367	0.0027	958.09	1382.41
DHRS9	-0.5467	0.0024	2796.1	1895.21		***PAQR9-AS1***	0.5233	0.0026	222.88	319.22
SPON2	-0.5433	0.0027	65838.19	44859.37		***ICAM5***	0.5167	0.0029	202.79	291.66
ABLIM3	-0.5433	0.0028	211.5	145.83						
WLS	-0.5433	0.0021	189.57	130.82						
LOC100507403	-0.54	0.0027	525.73	362.01						
AFAP1L1	-0.5267	0.0024	5020.69	3481.38						
CDKN2C	-0.5267	0.0025	743.57	514.08						
BCAR1	-0.5267	0.0025	145.64	101.13						
BUB1	-0.5233	0.0028	554.63	385.77						
BCAR1	-0.5167	0.0029	5901.39	4122.19						
GPSM2	-0.5133	0.0027	205.47	143.74						
